# Supplementary material for: Modeling glioblastoma heterogeneity as a dynamic network of cell states
Source: Mol Syst Biol. 2021 Sep 16;17(9):e10105. doi: 10.15252/msb.202010105 (PMC8444284; doi:10.15252/msb.202010105)
Supplement: Supplementary file 6 — Source Data for Figure 5 [file MSB-17-e10105-s004.zip › Figure5A_sourcedata/GSEA_3071/nosigdb_stateIII.GseaPreranked.1622795155562/RICHARDS_NOWAKOWSKI_SCIENCE_2017_OPC_UPREG.html]

Details for gene set RICHARDS\_NOWAKOWSKI\_SCIENCE\_2017\_OPC\_UPREG[GSEA]

|  || Dataset | state33071 |
| Phenotype | NoPhenotypeAvailable |
| Upregulated in class | na\_neg |
| GeneSet | RICHARDS\_NOWAKOWSKI\_SCIENCE\_2017\_OPC\_UPREG |
| Enrichment Score (ES) | -0.43552542 |
| Normalized Enrichment Score (NES) | -2.8906894 |
| Nominal p-value | 0.0 |
| FDR q-value | 0.0 |
| FWER p-Value | 0.0 |
Table: GSEA Results Summary

  

Fig 1: Enrichment plot: RICHARDS\_NOWAKOWSKI\_SCIENCE\_2017\_OPC\_UPREG      
 Profile of the Running ES Score & Positions of GeneSet Members on the Rank Ordered List

  

| PROBE | GENE SYMBOL | GENE\_TITLE | RANK IN GENE LIST | RANK METRIC SCORE | RUNNING ES | CORE ENRICHMENT || 1 | NDUFAF2 |  |  | 15 | 0.567 | 0.0031 | No |
| 2 | DSTN |  |  | 34 | 0.490 | 0.0045 | No |
| 3 | NME1 |  |  | 166 | 0.364 | -0.0258 | No |
| 4 | RPS27L |  |  | 188 | 0.348 | -0.0270 | No |
| 5 | ACP1 |  |  | 249 | 0.310 | -0.0390 | No |
| 6 | SEPT7 |  |  | 267 | 0.303 | -0.0397 | No |
| 7 | NDUFB3 |  |  | 312 | 0.288 | -0.0478 | No |
| 8 | DENR |  |  | 340 | 0.281 | -0.0515 | No |
| 9 | THY1 |  |  | 472 | -0.253 | -0.0831 | No |
| 10 | MBP |  |  | 493 | -0.254 | -0.0852 | No |
| 11 | ST3GAL5 |  |  | 519 | -0.255 | -0.0887 | No |
| 12 | PLOD2 |  |  | 521 | -0.255 | -0.0857 | No |
| 13 | HIPK2 |  |  | 546 | -0.257 | -0.0889 | No |
| 14 | NTM |  |  | 553 | -0.257 | -0.0873 | No |
| 15 | AFAP1L2 |  |  | 557 | -0.257 | -0.0848 | No |
| 16 | TMEM132B |  |  | 578 | -0.258 | -0.0869 | No |
| 17 | LITAF |  |  | 582 | -0.259 | -0.0844 | No |
| 18 | PHLDA1 |  |  | 589 | -0.259 | -0.0828 | No |
| 19 | DHCR24 |  |  | 598 | -0.260 | -0.0817 | No |
| 20 | ETV5 |  |  | 818 | -0.270 | -0.1365 | No |
| 21 | LRRTM3 |  |  | 827 | -0.271 | -0.1352 | No |
| 22 | EMC10 |  |  | 836 | -0.271 | -0.1339 | No |
| 23 | TM7SF3 |  |  | 852 | -0.272 | -0.1345 | No |
| 24 | BCHE |  |  | 867 | -0.272 | -0.1348 | No |
| 25 | KAT2B |  |  | 877 | -0.273 | -0.1338 | No |
| 26 | GNB4 |  |  | 885 | -0.274 | -0.1322 | No |
| 27 | IQSEC1 |  |  | 900 | -0.274 | -0.1325 | No |
| 28 | EVI2A |  |  | 902 | -0.274 | -0.1293 | No |
| 29 | TP53INP1 |  |  | 906 | -0.274 | -0.1266 | No |
| 30 | PDE1C |  |  | 933 | -0.276 | -0.1301 | No |
| 31 | TNRC6C |  |  | 940 | -0.276 | -0.1282 | No |
| 32 | SSR1 |  |  | 993 | -0.279 | -0.1385 | No |
| 33 | PHYHIPL |  |  | 1087 | -0.284 | -0.1597 | No |
| 34 | TMX3 |  |  | 1094 | -0.284 | -0.1577 | No |
| 35 | LPP |  |  | 1097 | -0.284 | -0.1547 | No |
| 36 | ATCAY |  |  | 1109 | -0.285 | -0.1540 | No |
| 37 | SLC44A1 |  |  | 1119 | -0.286 | -0.1528 | No |
| 38 | NOMO1 |  |  | 1158 | -0.288 | -0.1593 | No |
| 39 | SPATA6 |  |  | 1225 | -0.291 | -0.1732 | No |
| 40 | PLEKHH2 |  |  | 1282 | -0.294 | -0.1844 | No |
| 41 | RNF144A |  |  | 1311 | -0.295 | -0.1881 | No |
| 42 | PHLPP1 |  |  | 1317 | -0.296 | -0.1858 | No |
| 43 | MAMLD1 |  |  | 1352 | -0.298 | -0.1911 | No |
| 44 | ZNF516 |  |  | 1360 | -0.298 | -0.1892 | No |
| 45 | LRP1B |  |  | 1427 | -0.302 | -0.2029 | No |
| 46 | FGF14 |  |  | 1428 | -0.302 | -0.1991 | No |
| 47 | FIBIN |  |  | 1458 | -0.304 | -0.2030 | No |
| 48 | GRIA4 |  |  | 1488 | -0.305 | -0.2069 | No |
| 49 | ETV1 |  |  | 1495 | -0.306 | -0.2047 | No |
| 50 | ZEB1 |  |  | 1569 | -0.310 | -0.2202 | No |
| 51 | SPRY2 |  |  | 1592 | -0.311 | -0.2221 | No |
| 52 | DAG1 |  |  | 1598 | -0.312 | -0.2195 | No |
| 53 | MAML2 |  |  | 1603 | -0.312 | -0.2167 | No |
| 54 | DNM3 |  |  | 1676 | -0.317 | -0.2318 | No |
| 55 | SLITRK2 |  |  | 1725 | -0.320 | -0.2406 | No |
| 56 | SCD |  |  | 1737 | -0.321 | -0.2395 | No |
| 57 | NT5E |  |  | 1769 | -0.323 | -0.2437 | No |
| 58 | CPT1A |  |  | 1816 | -0.325 | -0.2518 | No |
| 59 | PTPRE |  |  | 1880 | -0.328 | -0.2644 | No |
| 60 | HIP1 |  |  | 1905 | -0.330 | -0.2667 | No |
| 61 | SPRY4 |  |  | 1970 | -0.334 | -0.2795 | No |
| 62 | FARP2 |  |  | 1971 | -0.334 | -0.2753 | No |
| 63 | CSGALNACT1 |  |  | 1976 | -0.334 | -0.2722 | No |
| 64 | PHLDB1 |  |  | 2001 | -0.335 | -0.2743 | No |
| 65 | LYPD1 |  |  | 2023 | -0.337 | -0.2757 | No |
| 66 | PCDH17 |  |  | 2043 | -0.338 | -0.2765 | No |
| 67 | TRIB2 |  |  | 2105 | -0.342 | -0.2884 | No |
| 68 | NLGN1 |  |  | 2107 | -0.342 | -0.2844 | No |
| 69 | PPIB |  |  | 2111 | -0.342 | -0.2809 | No |
| 70 | SCD5 |  |  | 2128 | -0.344 | -0.2808 | No |
| 71 | DSEL |  |  | 2180 | -0.347 | -0.2900 | No |
| 72 | CANX |  |  | 2209 | -0.349 | -0.2931 | No |
| 73 | SASH1 |  |  | 2231 | -0.351 | -0.2942 | No |
| 74 | ZNF385D |  |  | 2325 | -0.360 | -0.3145 | No |
| 75 | MAGI1 |  |  | 2340 | -0.361 | -0.3136 | No |
| 76 | TMEM206 |  |  | 2381 | -0.363 | -0.3197 | No |
| 77 | HAS2 |  |  | 2400 | -0.365 | -0.3199 | No |
| 78 | ARSB |  |  | 2416 | -0.366 | -0.3193 | No |
| 79 | LCORL |  |  | 2495 | -0.373 | -0.3354 | No |
| 80 | PDIA6 |  |  | 2505 | -0.373 | -0.3331 | No |
| 81 | ABAT |  |  | 2521 | -0.375 | -0.3323 | No |
| 82 | SCARB2 |  |  | 2697 | -0.390 | -0.3740 | No |
| 83 | XYLT1 |  |  | 2769 | -0.396 | -0.3879 | No |
| 84 | GPR155 |  |  | 2781 | -0.397 | -0.3858 | No |
| 85 | SEMA6D |  |  | 2812 | -0.400 | -0.3888 | No |
| 86 | ARNT2 |  |  | 2819 | -0.401 | -0.3853 | No |
| 87 | ALCAM |  |  | 2853 | -0.403 | -0.3890 | No |
| 88 | PDE4B |  |  | 2878 | -0.406 | -0.3903 | No |
| 89 | TENM1 |  |  | 2915 | -0.410 | -0.3947 | No |
| 90 | AASS |  |  | 2929 | -0.412 | -0.3930 | No |
| 91 | ITGAV |  |  | 2942 | -0.414 | -0.3910 | No |
| 92 | SLC22A17 |  |  | 2991 | -0.419 | -0.3985 | No |
| 93 | P2RX7 |  |  | 3016 | -0.422 | -0.3996 | No |
| 94 | ITGB8 |  |  | 3026 | -0.424 | -0.3966 | No |
| 95 | SGK1 |  |  | 3150 | -0.437 | -0.4239 | No |
| 96 | GPM6A |  |  | 3171 | -0.440 | -0.4236 | No |
| 97 | CHST3 |  |  | 3175 | -0.441 | -0.4189 | No |
| 98 | HSPA5 |  |  | 3193 | -0.442 | -0.4179 | No |
| 99 | MYO10 |  |  | 3196 | -0.442 | -0.4128 | No |
| 100 | PCDHGC3 |  |  | 3213 | -0.444 | -0.4115 | No |
| 101 | SEMA5A |  |  | 3229 | -0.447 | -0.4099 | No |
| 102 | CDCA7L |  |  | 3311 | -0.462 | -0.4256 | No |
| 103 | ITM2B |  |  | 3314 | -0.463 | -0.4203 | No |
| 104 | SLC2A13 |  |  | 3328 | -0.467 | -0.4179 | No |
| 105 | NTN1 |  |  | 3368 | -0.474 | -0.4223 | No |
| 106 | PCBP4 |  |  | 3381 | -0.476 | -0.4196 | No |
| 107 | PCDH9 |  |  | 3422 | -0.486 | -0.4241 | No |
| 108 | GALNT13 |  |  | 3466 | -0.497 | -0.4293 | Yes |
| 109 | CPEB2 |  |  | 3483 | -0.502 | -0.4272 | Yes |
| 110 | OLIG1 |  |  | 3486 | -0.503 | -0.4214 | Yes |
| 111 | LRRC4C |  |  | 3512 | -0.512 | -0.4217 | Yes |
| 112 | TSPAN7 |  |  | 3528 | -0.520 | -0.4191 | Yes |
| 113 | TNK2 |  |  | 3539 | -0.524 | -0.4152 | Yes |
| 114 | BAALC |  |  | 3542 | -0.525 | -0.4091 | Yes |
| 115 | CACNB2 |  |  | 3564 | -0.532 | -0.4080 | Yes |
| 116 | SERPINE2 |  |  | 3573 | -0.537 | -0.4034 | Yes |
| 117 | LRP1 |  |  | 3584 | -0.540 | -0.3993 | Yes |
| 118 | LAMA4 |  |  | 3587 | -0.542 | -0.3930 | Yes |
| 119 | ILDR2 |  |  | 3592 | -0.544 | -0.3872 | Yes |
| 120 | DST |  |  | 3597 | -0.545 | -0.3814 | Yes |
| 121 | PLAT |  |  | 3600 | -0.547 | -0.3751 | Yes |
| 122 | SPRY1 |  |  | 3605 | -0.552 | -0.3692 | Yes |
| 123 | SOX2 |  |  | 3613 | -0.555 | -0.3641 | Yes |
| 124 | ERBB3 |  |  | 3617 | -0.558 | -0.3579 | Yes |
| 125 | CHD7 |  |  | 3622 | -0.561 | -0.3519 | Yes |
| 126 | LSAMP |  |  | 3623 | -0.561 | -0.3448 | Yes |
| 127 | CTTNBP2 |  |  | 3627 | -0.563 | -0.3385 | Yes |
| 128 | CALCRL |  |  | 3631 | -0.565 | -0.3322 | Yes |
| 129 | CD9 |  |  | 3634 | -0.567 | -0.3256 | Yes |
| 130 | MAP2 |  |  | 3640 | -0.572 | -0.3198 | Yes |
| 131 | SHC3 |  |  | 3642 | -0.573 | -0.3128 | Yes |
| 132 | DUSP6 |  |  | 3648 | -0.577 | -0.3069 | Yes |
| 133 | FGFBP3 |  |  | 3649 | -0.578 | -0.2997 | Yes |
| 134 | RFTN2 |  |  | 3654 | -0.582 | -0.2934 | Yes |
| 135 | CDH13 |  |  | 3656 | -0.583 | -0.2863 | Yes |
| 136 | NLGN4X |  |  | 3666 | -0.589 | -0.2813 | Yes |
| 137 | SOX6 |  |  | 3675 | -0.596 | -0.2760 | Yes |
| 138 | IL1RAP |  |  | 3681 | -0.599 | -0.2698 | Yes |
| 139 | BMP7 |  |  | 3698 | -0.613 | -0.2663 | Yes |
| 140 | GRIA2 |  |  | 3721 | -0.633 | -0.2642 | Yes |
| 141 | SLC35F1 |  |  | 3724 | -0.635 | -0.2568 | Yes |
| 142 | FAM84A |  |  | 3729 | -0.639 | -0.2498 | Yes |
| 143 | SATB1 |  |  | 3732 | -0.644 | -0.2422 | Yes |
| 144 | VCAN |  |  | 3741 | -0.660 | -0.2361 | Yes |
| 145 | PCDH7 |  |  | 3745 | -0.665 | -0.2285 | Yes |
| 146 | CADM4 |  |  | 3751 | -0.673 | -0.2214 | Yes |
| 147 | MMP16 |  |  | 3753 | -0.675 | -0.2132 | Yes |
| 148 | HSP90B1 |  |  | 3757 | -0.681 | -0.2054 | Yes |
| 149 | ITPR2 |  |  | 3758 | -0.682 | -0.1968 | Yes |
| 150 | TNFRSF21 |  |  | 3763 | -0.691 | -0.1892 | Yes |
| 151 | TMEFF2 |  |  | 3767 | -0.697 | -0.1812 | Yes |
| 152 | LIMA1 |  |  | 3774 | -0.706 | -0.1740 | Yes |
| 153 | SULF2 |  |  | 3776 | -0.708 | -0.1653 | Yes |
| 154 | SALL3 |  |  | 3787 | -0.720 | -0.1590 | Yes |
| 155 | MAP3K1 |  |  | 3796 | -0.737 | -0.1518 | Yes |
| 156 | EDIL3 |  |  | 3810 | -0.758 | -0.1458 | Yes |
| 157 | LRRN1 |  |  | 3816 | -0.766 | -0.1375 | Yes |
| 158 | COL11A1 |  |  | 3828 | -0.788 | -0.1305 | Yes |
| 159 | WSCD1 |  |  | 3835 | -0.798 | -0.1220 | Yes |
| 160 | S100B |  |  | 3853 | -0.845 | -0.1159 | Yes |
| 161 | PROM1 |  |  | 3854 | -0.846 | -0.1053 | Yes |
| 162 | LRRN3 |  |  | 3859 | -0.856 | -0.0956 | Yes |
| 163 | KCNQ1OT1 |  |  | 3866 | -0.885 | -0.0861 | Yes |
| 164 | SCRG1 |  |  | 3869 | -0.904 | -0.0752 | Yes |
| 165 | PMP2 |  |  | 3876 | -0.959 | -0.0648 | Yes |
| 166 | C1orf61 |  |  | 3879 | -0.986 | -0.0529 | Yes |
| 167 | PTN |  |  | 3884 | -1.022 | -0.0411 | Yes |
| 168 | GPM6B |  |  | 3889 | -1.047 | -0.0290 | Yes |
| 169 | PTPRZ1 |  |  | 3913 | -1.342 | -0.0183 | Yes |
| 170 | PDGFRA |  |  | 3928 | -1.773 | 0.0003 | Yes |
Table: GSEA details [plain text format]

  

Fig 2: RICHARDS\_NOWAKOWSKI\_SCIENCE\_2017\_OPC\_UPREG: Random ES distribution      
 Gene set null distribution of ES for **RICHARDS\_NOWAKOWSKI\_SCIENCE\_2017\_OPC\_UPREG**
